# Fecal Volatile Organic Compounds in Preterm Infants Are Influenced by Enteral Feeding Composition

**DOI:** 10.3390/s18093037

**Published:** 2018-09-11

**Authors:** Sofia el Manouni el Hassani, Hendrik J. Niemarkt, Hager Said, Daniel J. C. Berkhout, Anton H. van Kaam, Richard A. van Lingen, Marc A. Benninga, Nanne K. H. de Boer, Tim G. J. de Meij

**Affiliations:** 1 Department of Pediatric Gastroenterology, Emma Children’s Hospital, Amsterdam UMC, Academic Medical Center, 1081 HV Amsterdam, The Netherlands; d.berkhout@vumc.nl (D.J.C.B.); m.a.benninga@amc.uva.nl (M.A.B.); 2 Department of Pediatric Gastroenterology, Emma Children’s Hospital, Amsterdam UMC, Vrije Universiteit Amsterdam, 1105 AZ Amsterdam, The Netherlands; h.said@vumc.nl (H.S.); t.demeij@vumc.nl (T.G.J.d.M.); 3 Neonatal Intensive Care Unit, Máxima Medical Center, 5504 DB Veldhoven, The Netherlands; Hendrik.Niemarkt@mmc.nl; 4 Neonatal Intensive Care Unit, Emma Children’s Hospital, Amsterdam UMC, Vrije Universiteit Amsterdam, 1081 HV Amsterdam, The Netherlands; a.h.vankaam@vumc.nl; 5 Neonatal Intensive Care Unit, Emma Children’s Hospital, Amsterdam UMC, University of Amsterdam, 1105 AZ Amsterdam, The Netherlands; 6 Neonatal Intensive Care Unit, Amalia Children’s Center/Isala, 8025 AB Zwolle, The Netherlands; r.a.van.lingen@isala.nl; 7 Department of Gastroenterology and Hepatology, Amsterdam UMC, VU University Medical Center, 1081 HV Amsterdam, The Netherlands; khn.deboer@vumc.nl

**Keywords:** VOC, volatile organic compound, eNose, nutrition, feces, breast milk, formula feeding, preterm infants, electronic nose, flatography

## Abstract

Fecal volatile organic compound (VOC) analysis has shown great potential as a noninvasive diagnostic biomarker for a variety of diseases. Before clinical implementation, the factors influencing the outcome of VOC analysis need to be assessed. Recent studies found that the sampling conditions can influence the outcome of VOC analysis. However, the dietary influences remains unknown, especially in (preterm) infants. Therefore, we assessed the effects of feeding composition on fecal VOC patterns of preterm infants (born at <30 weeks gestation). Two subgroups were defined: (1) daily intake >75% breastmilk (BM) feeding and (2) daily intake >75% formula milk (FM) feeding. Fecal samples, which were collected at 7, 14 and 21 days postnatally, were analyzed by an electronic nose device (Cyranose 320^®^). In total, 30 preterm infants were included (15 FM, 15 BM). No differences in the fecal VOC patterns were observed at the three predefined time-points. Combining the fecal VOC profiles of these time-points resulted in a statistically significant difference between the two subgroups although this discriminative accuracy was only modest (AUC [95% CI]; *p*-value; sensitivity; and specificity of 0.64 [0.51–0.77]; 0.04; 68%; and 51%, respectively). Our results suggest that the influence of enteral feeding on the outcome of fecal VOC analysis cannot be ignored in this population. Furthermore, in both subgroups, the fecal VOC patterns showed a stable longitudinal course within the first month of life.

## 1. Introduction

Early recognition and timely initiation of targeted treatment is a key prognostic factor in a wide variety of pediatric diseases, such as necrotizing enterocolitis (NEC), sepsis or (pediatric) inflammatory bowel disease (IBD). However, this is often hampered by a lack of accurate preclinical diagnostic biomarkers. Therefore, the search for novel, preferably non-invasive, diagnostic biomarkers remains necessary. An increasing number of studies have demonstrated the potential of fecal volatile organic compound (VOC) analysis as a diagnostic biomarker. VOC are the gaseous carbon-based organic chemicals and products of metabolic (patho-)physiological processes in the human body, which can be detected in various bodily excrements (e.g., exhaled breath, urine and feces). Fecal VOCs are considered to reflect the gut microbiota composition, function and interactions with the host [1,2]. Consequently, fecal VOC analysis is considered to be a powerful non-invasive diagnostic tool for gastrointestinal diseases, such as NEC and IBD, in which the intestinal microbiota alterations play an etiological role [3,4,5,6,7,8,9]. In addition, several studies have demonstrated the potential of fecal VOC analysis in diagnosing late-onset sepsis (LOS) and bronchopulmonary dysplasia (BPD) in preterm infants [10,11,12]. 

Different types of VOC analytical techniques are available, which can be divided into two different categories: (1) chemical analytical techniques and (2) electronic nose (eNose) devices. Chemical analytical techniques, such as gas chromatography–mass spectrometry (GC-MS), allow for the quantitative and qualitative detection of individual VOCs and is considered to be the gold standard in VOC detection. However, chemical analytical techniques tend to be time-consuming, expensive and labor intensive as it requires highly trained operating personnel and has high maintenance requirements, which thus limits its application as a clinical VOC detection method [13]. An alternative for chemical analytical techniques is the eNose device, which is based on VOC pattern recognition and thus is not able to quantify and identify specific VOCs. eNose devices enable real-time and high-throughput analysis; have inexpensive purchase and measurement costs; and produce relatively easy to interpret outcomes [13,14,15]. Due to the advantages of an eNose device, several studies have used an eNose device to assess its potential as a non-invasive diagnostic method and have produced positive outcomes in several diseases [6,8,10,11,16,17,18]. 

However, before VOC analysis can be applied as a diagnostic tool in daily clinical practice, substantial challenges, including methodological, biological and analytical problems, need to be managed. The effect of sampling and storage conditions and environmental influences on the outcome of VOC analysis have been reported in several studies [19,20]. For example, studies have demonstrated that fecal sample mass, water content, duration of storage at room temperature, fecal sample temperature and number of freeze-thaw cycles have a significant influence on the outcome of fecal VOC analysis. However, the influence of enteral feeding composition on the outcome of fecal VOC analysis, both in infants and adults, remains a significant gap in literature. This knowledge is pivotal for designing and interpreting studies that have suggests the potential of fecal VOC analysis as a noninvasive biomarker for NEC and sepsis in preterm infants. We hypothesized that the enteral feeding composition in preterm infants can influence fecal VOC profiles. In the current study, we aimed to compare fecal VOC profiles of preterm infants fed either breastmilk (BM) or formula milk (FM) by the means of an electronic nose (eNose) device (Cyranose 320^®^) in order to evaluate this potential influence. 

## 2. Materials and Methods

### 2.1. Subjects

The current study is part of an ongoing prospective multicenter study, including preterm infants born at a gestational age of ≤30 weeks and admitted at one of nine participating neonatal intensive care units (NICU) in The Netherlands and Belgium [5,6,7,10]. The aim of this study is to develop novel fecal biomarkers for NEC and sepsis. Fecal samples, clinical and demographic data were collected daily from birth up to 28 days postnatally. Clinical and demographic data included birth weight, gestational age, mode of delivery, type of enteral feeding, age achieving full enteral feeding, clinical condition and medication usage. No probiotic administration was administrated routinely in the participating centers. 

For the current study, healthy preterm infants born at a gestational age ≤30 weeks were eligible to participate. Infants were born during the period of October 2014 and January 2018 in four of nine participating centers: Emma Children’s Hospital (location Vrije Universiteit Amsterdam (VUmc) and Academic medical center (AMC), both situated in Amsterdam), Amalia Children’s Clinic (Zwolle) and Máxima Medical Center (Veldhoven). We chose to include infants of only four of nine available centers in order to limit the influence of center-specific variation in VOCs [19]. These specific centers were selected based on the number of available samples for further analysis. Infants were excluded in the case of NEC, spontaneous intestinal perforation (SIP), early and late onset sepsis (EOS and LOS) within the inclusion period and if the collected fecal mass was insufficient for VOC analysis. In this study, preterm born infants suffering from NEC, SIP, EOS and LOS were excluded since the studies have demonstrated differences in fecal VOC composition in these diseases [3,4,6,9,10,12]. Study approval was provided by the local institutional review boards of all participating centers (amendment A2016.363). Written informed consent was obtained from both parents of all included infants. 

### 2.2. Study Groups

Infants were divided into two subgroups based on enteral feeding composition postnatally: (1) BM fed, which was defined as >75% of the total daily enteral feeding volume consisting of BM and (2) FM fed, which was defined as >75% of the total daily enteral feeding volume consisting of FM. Included infants had to fulfill these criteria on each of the first 28 days, except on the first three days of life, since the volume and ratio of BM and FM was highly variable in this period. We decided to use these criteria since none of the infants were administered exclusively FM after the first three days, especially due to the feeding policy in The Netherlands that promotes BM. In Emma Children’s Hospital (at both locations), donor milk was administered when the milk from the infant’s own mother was insufficient, which was considered to be an alternative for BM. In this study, only human donor milk and FM were pasteurized prior to administration. A neonate was considered to be fully enteral fed when parenteral feeding was ceased for two consecutive days. Infants from both groups were strictly matched based on center of birth, birth weight, gestational age and total days of antibiotic exposure within the inclusion period. 

### 2.3. Sample Collection

Fecal samples were collected daily from the diaper by a nurse from birth up to 28 days postnatally. As stated above, the current study is part of a large ongoing study in which the fecal samples are collected daily up to a postnatal age of 28 days since both NEC and LOS tend to occur in the first month of life [21,22]. Therefore, in the current study, a follow up of 28 days for the fecal sample collection is chosen. In addition, preterm infants are most likely to be fully enteral fed within the first two weeks of life [23]. The analysis of fecal samples derived in the first month of life provides us with a good representation of the population. Fecal samples were collected in a sterile stool container (Stuhlgefäß 10 mL, Frickenhausen, Germany) and immediately stored at −20 °C until further handling. In the case of multiple stool productions per day, only the first fecal sample was stored. Sample collection was ceased prematurely when infants were transferred, discharged or died before the postnatal age of 28 days. Fecal samples collected at postnatal day 7, 14 and 21 (±1 day) postnatal were used for fecal VOC analysis. 

### 2.4. VOC Analysis by eNose

In the current study, the samples were analyzed by means of an eNose device (Cyranose 320^®^, Smiths Detections, Pasadena, CA, USA). The Cyranose 320^®^ is a portable chemical vapor analyzer containing a nanocomposite array, which consists of 32 individual thin-film carbon-black polymer sensors. Exposure to VOCs results in the competitive interaction of these polymer sensors and swelling, consequently changing electrical resistance. Due to the unique polymer coating of each sensor, a different change in electrical resistance is registered upon contact with the gaseous VOC mixture. A combination of the individual outcome of electrical resistance is combined with one VOC pattern for each sample. The differentiation between two study groups is based on pattern recognition analysis [6,7,10,11]. 

Approximately 150 mg of the fecal sample (±10% with a minimum of 100 mg) was transferred from the stored fecal sample into a sealed vacutainer (BD vacutainer, Belliver Industrial Estate, Plymouth, UK). Samples were weighed on a calibrated scale (Mettler Toledo, AT 261 Delta Range, Columbus, OH, USA) and the vacutainers were resealed. Prior to analysis, the samples were thawed to room temperature (18 °C) for 10 min. Subsequently, the vacutainers were connected to the eNose in an air-tight closed loop system to prevent headspace (3 mL) dilution. This system was created by piercing two needles (Terumo Europe N.V., Leuven, Belgium) through the cover top of the container and connecting this with the eNose by a tube (Argyle Kendall tube 3 mm, Mansfield, MA, USA). A polyethersulfone syringe water filter (VWR International B.V., Arlington Heights, IL, USA) and a 3-way stopcock system (BD Connecta, Helsinborg, Sweden) were included in the system to control the airflow direction and prevent contamination of the eNose by condensation. The analysis of the fecal samples were performed at room temperature without preheating the samples. A stable baseline reference signal was created by connecting a VOC-filter (A1, North Safety, Middelburg, The Netherlands) to the eNose. Subsequently, the headspace was sampled for 60 s in order to reach a stable reference sensor response. After the sample analysis, all sensors were purged with VOC-filtered air to wash off the VOCs and create a stable baseline for the subsequent samples (control value). In addition, blank measurements were performed prior to every sample measurement by using an empty vacutainer. Needles, hoses and 3-way stopcocks were replaced after each sample measurement. The analysis time of the eNose was approximately 180 s. All samples were analyzed randomly in two subsequent days, consisting of four measurement sessions of approximately 3 h.

### 2.5. Statistical Analysis

#### 2.5.1. Demographic and Clinical Data

Statistical analysis was performed using Statistical Package for the Social Science (SPSS) version 22.0. Demographic and clinical data were compared by an independent *t*-test, Mann-Whitney test or Chi-Square test as appropriate. Normal distributed continuous data are presented as mean and standard deviation. Non-normally distributed continuous data are presented as median and interquartile range (IQR). Categorical data are presented as counts and percentages. Results were considered significant at a *p*-value < 0.05. 

#### 2.5.2. eNose Data

In order to reduce the risk of overfitting the diagnostic algorithm, the principal component analysis (PCA) was performed on the raw data output of the eNose. This analysis allows for the recombination of the variance of the original dataset into a set of orthogonal principle components or factors. For each predefined time-point, the principle components that differentiate the two subgroups (e.g., BM or FM fed infants) were selected by the means of a Student *t*-test (*p*-value < 0.05). The two most significant PCs were subsequently used in a supervised canonical discriminant analysis, which was internally validated by a leave one-out method in view of the relatively small sample size. This method builds a diagnostic algorithm using data from all but one of the subjects. The remaining subject is subsequently introduced to the algorithm and classified, which provides a probability of enteral feeding composition for that case. This repetitive process was repeated until each subject has been excluded from the primary algorithm once. A combination of probabilities for all cases was used to construct a receiver operator characteristic curve (ROC) and compute the single point sensitivity and specificity values. The overall accuracy of the algorithm was assessed by the area under the curve (AUC) and related 95% confidence interval [6,7,10,11]. 

Secondly, by the means of linear mixed model analysis changes over time were assessed for both study groups by using the PCs of all three time-points. 

## 3. Results

### 3.1. Patient Population

**Figure 1:**
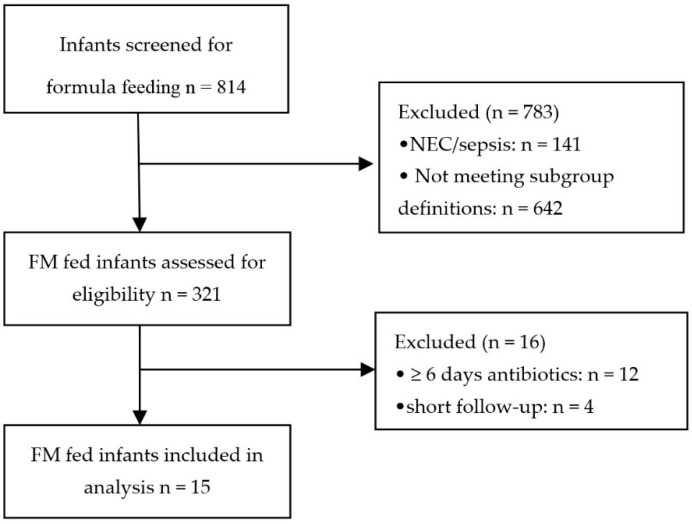
Flowchart inclusion process for formula milk fed infants. Abbreviations: NEC = necrotizing enterocolitis; FM = formula milk; and *n* = number.

Fecal samples from 30 preterm infants (15 FM fed, 15 BM fed) were analyzed by the means of an eNose (Cyranose 320^®^). In Figure 1, the inclusion process is displayed. The median gestational age was 29 weeks for the FM fed infants and 29 weeks plus one day for the BM fed infants. The median sample storage time at −20 °C for the samples of FM fed infants was 28 months and 26 months for the samples of BM fed infants. No significant differences were seen between the groups for the variables of age, gender, birth weight, gender, center, delivery mode, age at full enteral feeding, sample mass, sample age, total days of parental feeding and parental feeding types, antibiotics administration and medication administration (Table 1). In addition, both groups received the same amount of additives, such as protein fortifiers, in their enteral feeding. 

### 3.2. VOC Analysis

**Figure 2:**
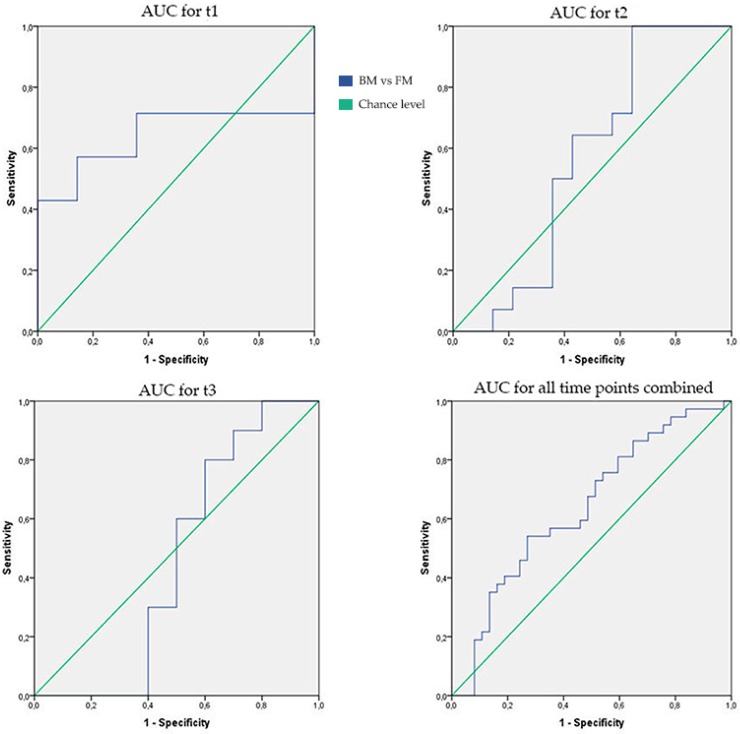
Area under curve (AUC) for all individual time-points and all time-points combined.

**Figure 3:**
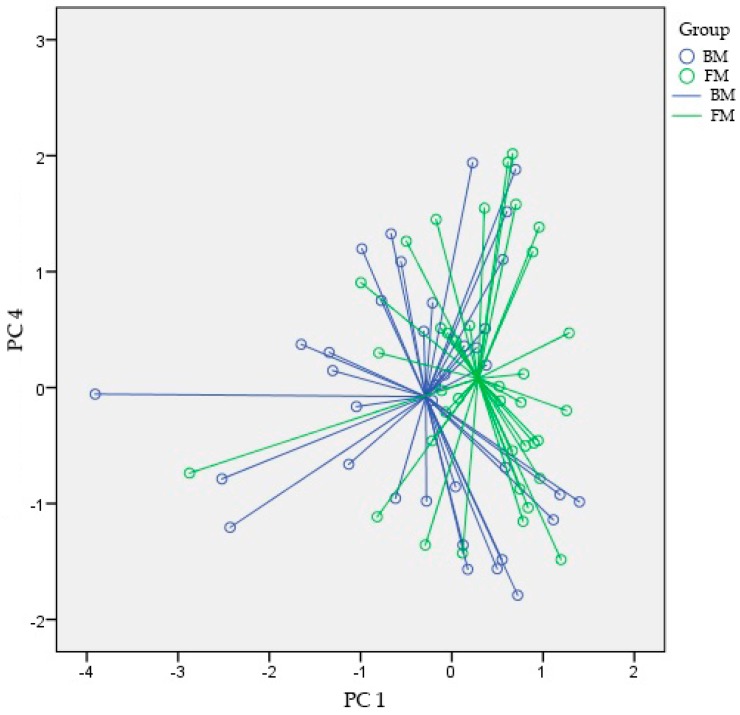
Scatterplot of the best performing principal components for all time-points combined. Abbreviations: BM = breastmilk; FM = formula milk; and PC = principal component.

There were no significant differences between the fecal VOC patterns of BM and FM fed preterm infants at t1 (AUC [95% CI]; *p*-value; sensitivity; and specificity of 0.64 [0.41–0.87]; 0.20; 71%; and 64%, respectively), t2 (0.56 [0.33–0.79]; 0.58; 64%; and 57%) and t3 (0.46 [0.18–0.74]; 0.76; 60%; and 50%) (Figure 2 and Table 2). Since no statistically significant differences in VOC profiles within both groups over time (t1 to t3) were seen (PC 1 *p*-value = 0.49; PC2 *p*-value = 0.12; PC3 *p*-value = 0.71; and PC4 *p*-value = 0.97), we performed a post-hoc analysis by pooling the fecal VOC profiles from all measured time-points. Here, we observed a statistically significant difference between both subgroups although the overall accuracy was modest (0.64 [0.51–0.77]; 0.04; 68%; and 51%) (Figure 2). Figure 3 shows a scatter plot for the two most statistically significant PCs (PC1 and PC4) for all time-points combined for the two study groups. 

## 4. Discussion

In the present study, we have compared the VOC profiles of preterm infants with two different feeding strategies (BM versus FM fed) at three time-points within the first month of life. No differences were found in the fecal VOC profiles between the BM and FM fed preterm infants at the predefined time-points. However, when the fecal samples from all time-points were pooled, a statistically significant difference was observed between the VOC profiles of both subgroups, indicating that enteral feeding composition influences VOC profile outcomes. 

Various studies have demonstrated the potential of VOC analysis as a non-invasive diagnostic biomarker for NEC, LOS and BPD [6,9,10,11,12]. In both NEC and LOS, studies have demonstrated the differences in VOC profiles between healthy controls and cases, which occurs up to three days prior to clinical onset. Furthermore, in a pilot study of Garner et al., NEC-specific fecal VOCs were identified, which highlights its potential in the development of disease-specific sensors to be used in clinical practice [9]. 

We found no differences in the fecal VOC profiles at predefined time-points between BM and FM fed infants. However, after pooling the profiles of different time-points, thereby increasing the number of inclusions per analysis, a statistically significant difference was found between both subgroups. A possible explanation for this apparent discrepancy is that feeding types are only weakly associated with specific VOC signatures, which even may be masked by the commonly high inter-individual variability within this population. By enlarging the number of samples, the relatively small VOC differences between both groups may have become statistically significant. This finding suggests that the influence of dietary intake on the outcome of fecal VOC analysis cannot be ignored in this specific population. Consequently, in future studies focusing on fecal VOC analysis, dietary intake must be included in the matching procedure or should be corrected for at least in this specific population. The difference in VOC profiles between BM and FM fed infants may be explained by the feeding-associated microbial differences. Recent studies have demonstrated a higher initial diversity of gut microbiota communities in preterm BM fed infants compared to FM fed infants, which may have resulted in the simultaneous differences in VOC profiles [24,25,26]. Furthermore, the compositional differences between BM and FM itself could explain the differences in VOCs between both groups. VOCs derived from formula milk predominantly contain alcohols, aldehydes and ketones, while VOCs from BM mainly contain secondary oxidation products and terpenes [27]. These differences in VOCs could have contributed to the observed differences in fecal VOC patterns. 

In the present study, no differences in the VOC profiles over time (from t1 to t3) were found within and between both subgroups, indicating that fecal VOC profiles have a fairly stable longitudinal course in the first month of life. Similarly, only a gradual shift in microbiota composition is seen within the first 30 days of life, while a fairly stable microbiota composition is achieved around the first year of age [25,28,29]. Another study demonstrated that in formula-fed infants, higher initial and persistent levels of *Bacillales/Lactobacillales* seem to delay colonization by other taxa when compared to BM fed infants [26]. In FM fed infants, *Enterobacteriales* was not observed until an adjusted gestational age of 34 weeks, while BM fed infants had high levels of *Enterobacteriales* from the 29th week of postmenstrual age onwards. Furthermore, a more rapid increase in *Clostridiales* in the BM fed group was observed compared to the FM fed group [26]. A systematic review on the pathogen specific VOCs reported the presence of VOCs that are produced by more than one bacterial strain [30]. Unfortunately, no specific VOC profiles for *Enterobacteriales* and *Lactobacillales* are described. However, theoretically, there can be a significant number of overlapping fecal VOCs for these bacteria, which cannot be distinguished by means of an eNose device. 

VOC analysis by an eNose device is based on pattern recognition rather than on the identification of individual volatile molecules, which can be obtained through GC-MS. Thus far, most studies on fecal VOC analysis in preterm infants have made use of an eNose due to several advantages, including its low cost, easy use and high throughput capacities. In order to assess which unique (set of) VOCs are responsible for the observed differences, GC-MS analysis would be indicated. In addition, the specific VOCs responsible for the difference in the outcome of VOC analysis between both feeding types should be considered in the development of disease-specific sensors. Therefore, current study results should be confirmed by conducting a study in which the fecal VOC composition is compared between BM and FM fed preterm infants by means of GC-MS.

This study has several strengths. In all cases, the demographic and detailed clinical features were collected prospectively and the samples were collected and stored in a standardized matter. For this study, only healthy infants were included to circumvent bias by the measurement of disease-specific VOCs. In addition, infants were strictly matched based on gestational age, center, birth weight and antibiotic usage, which are all factors that have been considered to influence the outcome of VOC analysis [31,32]. Since the majority of NICUs in The Netherlands aim to administer either BM or human donor milk, only a limited amount of healthy preterm infants receive >75% of formula milk. We limited the number of participating centers (*n* = 4) since a previous study demonstrated a significant influence of center on the outcome of VOC analysis, which is possibly due to the usage of different protocols [19]. 

There are some limitations of this study that need to be considered. In the first weeks of life, the preterm infants commonly receive a combination of FM and BM in different ratio and therefore, we chose to include infants with a cutoff point of 75% of total volume of either BM or FM on a daily basis. Since the number of infants receiving completely formula milk was limited, it could be hypothesized that the differences in fecal VOC profiles of FM and BM fed infants might actually be larger than observed in the current study. Another limitation is that the potential VOC-differences between the different types of formula feeding could not be assessed since this subgroup was too small to perform formula-specific analysis. Lastly, studies have indicated that the prolonged storage of fecal samples in the freezer might result in a loss of VOC diversity. Therefore, only samples with a storage time of less than two and a half years were included. Storage time was equally distributed in both groups so we consider the risk of a type I error by differences in storage time to be limited. 

## 5. Conclusions

In conclusion, no differences in the fecal VOC profiles between BM and FM fed preterm infants were observed at the separate postnatal time-points of days 7, 14 and 21. When increasing the number of inclusions per analysis by combining the outcome of VOC analysis from these time-points, a statistically significant difference was noticed between both groups. Although the accuracy to discriminate both groups was only modest, our results suggest that the influence of dietary intake on the outcome of fecal VOC analysis cannot be ignored in this specific population and should therefore be considered when interpreting VOC data. Furthermore, a stable longitudinal course within the first month of life is demonstrated for fecal VOC patterns both in healthy BM and FM fed infants, which is similar to the fairly stable course of microbiota composition described in previous studies. 

## Figures and Tables

**Table 1:** Demographics patient population.

	Formula Fed Infants (*n* = 15)	Breastmilk Fed Infants (*n* = 15)	*p*-Value
Gestational age (median [IQR], weeks + days)	29 + 0 [28 + 1–29 + 6]	29 + 1 [28 + 2–29 + 4]	0.723
Birth weight (median [IQR], g)	1200 [1030–1280]	1220 [1005–1285]	0.740
Gender Male (*n*[%])	10 [66.7]	8 [53.3]	0.464
Delivery mode Vaginal delivery (*n*[%])	5 [33.3]	7 [46.7]	0.464
Age at full enteral feeding (median [IQR] days)	10 [8–12]	9 [8–15]	0.964
Parental feeding (median [IQR], days)	8 [7–10]	8 [6–13]	0.893
t1	9	9	
t2	0	2	
t3	0	0	
Type of parental feeding (*n*[%])			
Lipids	3 [20]	3 [20]	0.878
Combination Lipids and Proteins	3 [20]	3 [20]	
Intralipid 20% + vaminolact-glucose	1 [6.7]	2 [13.3]	
Smofolipid + vaminolact-glucose	2 [13.3]	1 [6.7]	
Enteral additives (*n*[%])			
None	8 [53.3]	2 [13.3]	0.073
MT	1 [6.7]	5 [33.3]	
PF	1 [6.7]	0 [0]	
MT and PF	0 [0]	1 [6.7]	
BMF	4 [26.7]	7 [46.7]	
Amino acid formula	1 [6.7]	0 [0]	
Received donor milk (*n*[%])	2 [13.3]	0 [0]	0.150
Sample masses (median [IQR] in g)			
t1 (*n* = 14)	0.150 [0.148–0.151]	0.151 [0.150–0.153]	0.211
t2 (*n* = 14)	0.150 [0.148–0.153]	0.150 [0.148–0.152]	0.780
t3 (*n* = 10)	0.151 [0.150–0.152]	0.151 [0.150–0.153]	0.538
Sample age (median (IQR], in months)			
t1 (*n* = 14)	28 [16.5–38]	26 [18–36]	0.913
t2 (*n* = 14)	27.5 [16.5–38]	25.5 [18–36]	0.836
t3 (*n* = 10)	27.5 [15–38]	24.5 [18–37]	0.850
Postpartum antibiotics (*n*[%])			0.513
Not administered	1 [6.7]	0	
1–3 days administered	13 [86.7]	13 [86.7]	
>3 days administered	1 [6.7]	2 [13.3]	
Antibiotic exposure (*n*[%])	15 [100]	15 [100]	1.000
Antibiotics days (median (IQR])	2 [1–2]	2 [1–2]	0.128
Medication (*n*[%])			
Antiviral medication	2 [13.3]	0	0.150
Caffeine	14 [93.3]	15 [100]	0.753
Vitamin D	9 [60]	9 [60]	1.000
Vitamin E	3 [20]	2 [13.3]	0.630
Iron supplementation	4 [26.7]	6 [40]	0.446
Morphine	3 [20]	0	0.073
Propofol	2 [13.3]	0	0.150
Paracetamol	1 [6.7]	1 [6.7]	1.000
Ibuprofen	1 [6.7]	1 [6.7]	1.000
Indomethacin	0	1 [6.7]	0.317
Chloral hydrate	0	1 [6.7]	0.317
Nystatin	0	2 [13.3]	0.150
Miconazole	3 [20]	1 [6.7]	0.291
Heparin	1 [6.7]	0	0.317
Dopamine	2 [13.3]	0	0.150
Dobutamine	1 [6.7]	0	0.317
Hydrochlorothiazide	2 [13.3]	0	0.150

Abbreviations: IQR = Interquartile range; MT = Milk thickener; PF = protein fortifier; BMF = breastmilk fortifier; TPN = total parental nutrition; *n* = number; and g = grams.

**Table 2:** Results fecal VOC analysis on different time points.

	AUC [95% CI]	*p*-Value	Sensitivity	Specificity
**t1**	0.64 [0.41–0.87]	0.20	71%	64%
**t2**	0.56 [0.33–0.79]	0.58	64%	57%
**t3**	0.46 [0.18–0.74]	0.76	60%	50%
**All time points combined**	0.64 [0.51–0.77]	0.04	68%	51%

Abbreviations: t = time point; AUC = Area under curve; and CI = confidence interval.
